# A Case of Moral Insanity, Caused by a Depression of the Skull, Cured by an Operation

**Published:** 1846-10

**Authors:** 


*A Case of Moral Insanity, caused by a depression of the Skull, cured by an operation.*—The following case, related by Dr. Robertson, in the *Northern Journal,* illustrates some interesting points in the physiology of the brain, as well as in the causes and treatment of insanity :

"Robert Driver, aged twenty-three, a sailor, was admitted into the Dunstan Lodge Asylum, in February, 1845.

"Ten years previously, he fell from the mast of a ship; the fall was followed by an attack of acute mania. On his return home he became more and more ungovernable in his temper, and violent in his conduct. He also suffered from frequent pains in the part of the cranium on which he fell, and which he imagined were caused by his mother beating him.

"After being some time in this asylum, this delusion gave way, and the intellectual powers of his mind remained sound ; but his conduct continued ungovernable, and his language abusive, and kind words made no impression on his wayward temper. He still complained of pains in the injured part. On examining his head, a very distinct depression was discovered on the posterior superior margin of the right parietal bone, the situation to which he referred the pains.

"In consultation with Mr. Furness of Newcastle, consulting surgeon to this institution, it was decided that the depressed portion of skull be removed by the trephine.

"On the 3d of January, the operation was skilfully performed by Mr. Furness. The patient bore it well, and the wound healed without a bad symptom. The portion of the cranium removed was healthy in appearance on both of its surfaces. It adhered very firmly to the dura mater, requiring considerable force for its removal. It was altered considerably in form, appearing to have been indented, rather than fractured, which is not improbable, seeing the accident occurred to the patient when only thirteen years of age.

"His conduct is now, and has been since the operation, in every way improved. He has had no bursts of passion ; answers civilly when spoken to, and is grateful for the relief afforded him. He looks forward with pleasure to his return home, which will take place as soon as the weather improves. He has for the last fortnight been working in the farm, and states, that since the operation he has been free from pain in the head, under which he formerly laboured." —*Lancet.*

